# Risk Factors for the Failure of Non-surgical Treatment in Pneumothorax

**DOI:** 10.7759/cureus.97132

**Published:** 2025-11-18

**Authors:** Waku Nakano, Tomoaki Nakamura, Atsushi Kitamura, Clara So, Shosei Ro, Ryosuke Imai, Kohei Okafuji, Yutaka Tomishima, Torahiko Jinta, Naoki Nishimura

**Affiliations:** 1 Department of Pulmonary Medicine, St. Luke’s International Hospital, Tokyo, JPN

**Keywords:** chest tube drainage, interstitial lung disease, pleurodesis, pneumothorax, treatment failure

## Abstract

Introduction: Few studies have thoroughly investigated the factors influencing the clinical course of pneumothorax at each stage of treatment. This study aimed to address this gap by examining these factors.

Methods: We conducted a single-center retrospective study involving patients with pneumothorax aged 20 years or older admitted to our hospital. Patients were divided into two groups: those successfully treated with chest tube drainage alone (Group A) and those who required additional treatment (Group B). Excluding patients requiring surgical treatment, patients in Group B were subsequently divided into two groups: those successfully treated with non-surgical secondary treatments (Group C) and those who were not (Group D). Clinical factors were compared between Groups A and B and between Groups C and D.

Results: Three hundred and ninety-five patients were included in this analysis. A comparison between Group A (148 patients) and B (247 patients) revealed significant differences in the presence of interstitial lung disease (ILD) (6.1% vs. 13.8%, *p* < 0.05) and pneumothorax history (20.4% vs. 42.5%, *p* < 0.05). Among patients in Group B, 172 underwent surgery as the next treatment, while 75 received non-surgical treatment after chest tube drainage. Significant differences were observed between Group C (27 patients) and D (48 patients) in heavy smoking history (33.3% vs. 62.5%, *p* < 0.05) and presence of ILD (25.9% vs. 52.1%, *p* < 0.05).

Conclusion: Patients with a history of ILD are more likely to require additional treatment after chest tube drainage.

## Introduction

In cases of clinically significant pneumothorax requiring intervention, a chest tube is placed for drainage. If the air leak persists, additional interventions are considered; when surgery is not feasible, alternative treatments such as pleurodesis, endobronchial valves, or endobronchial Watanabe spigot (EWS) may be employed [1,2]. Patients with poor cardiopulmonary reserve, including those with interstitial lung disease (ILD) and chronic obstructive pulmonary disease (COPD), often present with inoperable cases, making secondary pneumothorax in these conditions particularly challenging to manage [3-5]. However, few studies have examined the factors influencing treatment at various stages, including during chest tube placement or non-operative approaches in inoperable cases. Therefore, this study aimed to identify patients unlikely to be successfully treated with chest tube drainage alone and to determine the factors contributing to the failure of non-surgical treatment in inoperable cases. Additionally, the success rate of each nonoperative treatment was evaluated.

## Materials and methods

Patients

We conducted a single-center, retrospective study of patients aged 20 years or older who were admitted to our hospital with pneumothorax between January 1, 2011, and March 31, 2022. Patients with traumatic or medically induced pneumothorax, those without chest tube drainage, those transferred to other hospitals, and those who died on the day of admission were excluded from the study. The study was approved by the Institutional Ethics Committee of St. Luke’s International Hospital (approval number: 22-J013, approval date: August 18, 2022). Procedures followed were in accordance with the Declaration of Helsinki.

Patients were divided into two groups: those with a pneumothorax that resolved with chest tube drainage alone (Group A) and those with a pneumothorax that did not resolve with chest tube drainage alone (Group B). After excluding patients who underwent surgery as a secondary treatment from Group B, the remaining patients were further classified into those with a pneumothorax that resolved with non-surgical secondary treatments (Group C) and those with a pneumothorax that did not resolve with non-surgical secondary treatments (Group D). Non-surgical treatments included pleurodesis, autologous blood patches, EWS placement, drain tube replacements, or additional drain tube insertions.

Clinical factors were compared between Groups A and B and between Groups C and D. Variables assessed included age, sex, history of heavy smoking (30 pack-years or more), serum albumin level, comorbidities (COPD, ILD, or history of pneumothorax), treatment type, and length of hospital stay, all of which were obtained from previous medical records. “Resolution” was defined as “the absence of air accumulation in the pleural cavity on chest radiography within 24 hours of chest tube removal,” whereas “no resolution” was defined as a prolonged air leak lasting more than 48 hours. Heavy smoking history was defined as 30 pack-years or more, in accordance with the definition of the Lung Cancer Screening Guidelines 2022 of the Japanese Lung Cancer Society [6]. Data on COPD and ILD were extracted from the patient’s medical histories. The non-surgical success rate was calculated as the proportion of resolutions achieved relative to the total number of treatments administered at each stage (first, second, third, fourth, etc.). For example, if a single patient was treated with autologous blood, EWS, and OK-432, each was counted as a separate treatment. The analysis included two groups of patients: all patients who received non-surgical secondary treatment and those with a pneumothorax that did not resolve with non-surgical secondary treatments.

Pneumothorax treatment practice

The indication for surgery was determined by the surgeon upon admission. Generally, non-surgical treatments, typically autologous blood or OK-432 for pleurodesis, were initially performed, with the final treatment decision determined by the treating physician. Owing to insurance restrictions, talc was not used until March 2022. In cases of incomplete lung expansion, the attending physician decided whether to add or replace the tube or perform EWS.

Statistical analysis

Categorical variables were compared using Fisher’s exact test, while continuous variables were compared using the Wilcoxon rank-sum test (equivalent to the Mann-Whitney U test; we report the W statistic). To identify risk factors for treatment failure, a multivariable logistic regression model was used, including potential risk factors such as age, sex, serum albumin level, presence of ILD, pneumothorax history, and heavy smoking history (30 pack-years or more). The significance level was set at *p* = 0.05. All statistical analyses were performed using EZR (Saitama Medical Center, Jichi Medical University, Saitama, Japan), a graphical user interface for R (The R Foundation for Statistical Computing, Vienna, Austria, version 4.0.4) [7].

## Results

**Figure 1:**
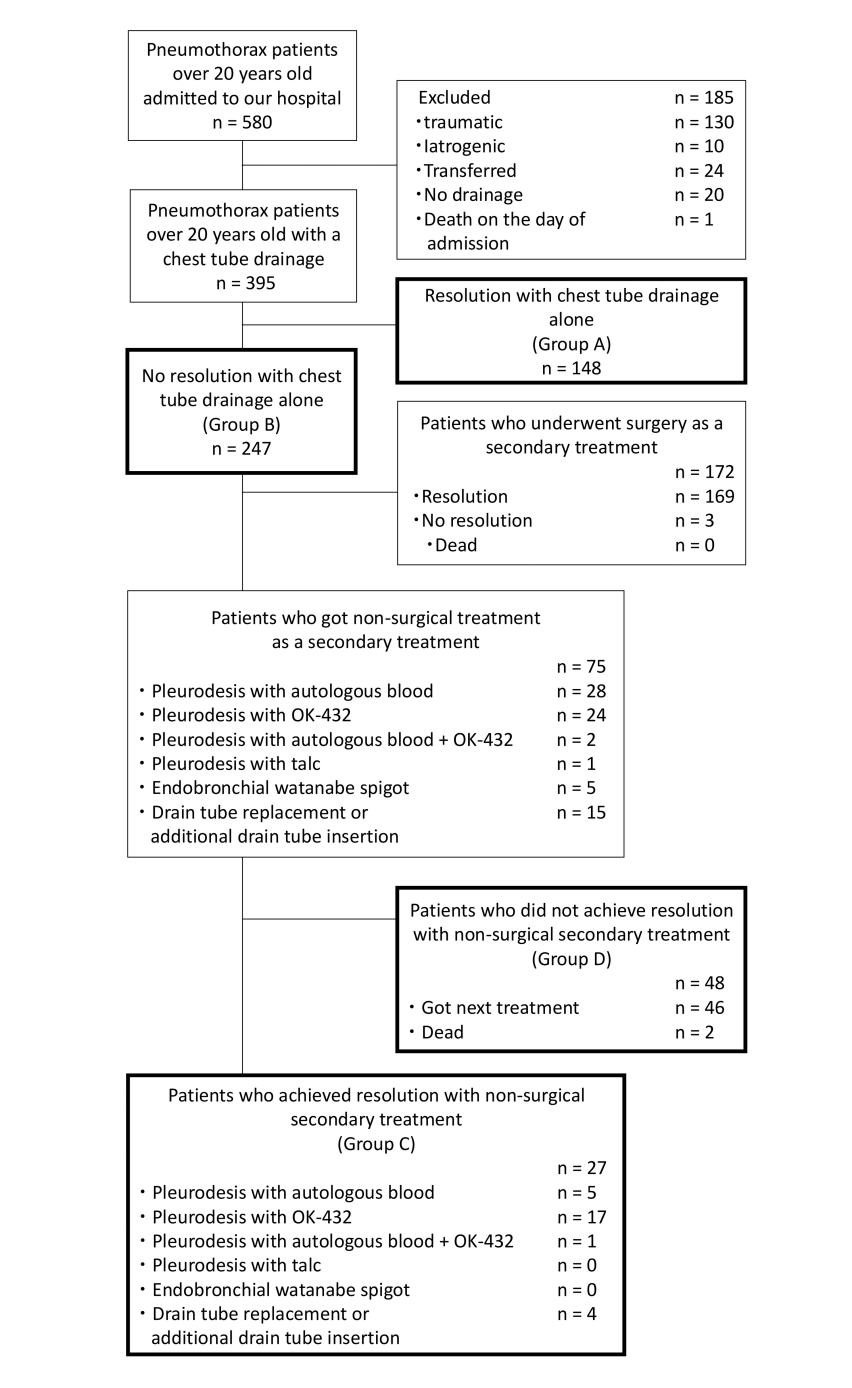
Target patients

Of 580 patients aged over 20 years admitted with pneumothorax to our hospital, 185 were excluded, and 395 were included in the analysis (Figure 1).

Among these, 148 patients had resolution of pneumothorax with chest tube drainage alone (Group A), whereas 247 patients required additional treatment (Group B). Patients in Group B were more likely to have ILD (9 [6.1%] vs. 34 [13.8%], *p* < 0.05) and pneumothorax history (30 [20.4%] vs. 105 [42.5%], *p* < 0.05) (Table 1).

**Table 1:** Characteristics of patients with pneumothorax treated with chest tube drainage Abbreviations: COPD, chronic obstructive pulmonary disease; ILD, interstitial lung disease.

	Resolution with chest tube drainage alone (Group A) n = 148	No resolution with chest tube drainage alone (Group B) n = 247	Test	Statistic	p-value
Age, median (range), years	48 (30–70)	40 (29–65)	Wilcoxon rank-sum	W = 19979	0.121
Male, n (%)	119 (80.4)	210 (85.0)	Fisher’s exact	—	0.265
>30 pack years, n (%)	35 (23.6)	60 (24.3)	Fisher’s exact	—	0.904
Serum albumin, median (range), mg/dL	4.3 (4.1–4.7)	4.4 (3.9–4.7)	Wilcoxon rank-sum	W = 18188	0.935
Comorbidity, n (%)					
COPD	18 (12.2)	29 (13.0)	Fisher’s exact	—	0.877
ILD	9 (6.1)	34 (13.8)	Fisher’s exact	—	0.019
History of pneumothorax, n (%)	30 (20.4)	105 (42.5)	Fisher’s exact	—	<0.001

The multivariate analysis revealed significant differences in age, ILD history, and pneumothorax history (Table 2).

**Table 2:** Multivariable logistic regression analysis of factors associated with resolution using chest tube drainage alone Abbreviations: OR, odds ratio; CI, confidence interval; ILD, interstitial lung disease.

	OR	95% CI	p-value
Age	0.979	0.965–0.992	0.002
Male	0.665	0.373–1.190	0.167
>30 pack years	1.290	0.703–2.350	0.414
Serum albumin	0.797	0.516–1.230	0.307
ILD	4.010	1.690–9.500	0.002
History of pneumothorax	2.910	1.790–4.720	<0.001

In Group B, 172 patients underwent surgery as the next treatment after chest tube placement, while 75 received non-surgical treatment after drain placement. Of these 75 patients, 27 achieved resolution with a single non-surgical intervention (Group C), whereas 48 required multiple non-surgical interventions (Group D). Patients in Group D were significantly more likely to have ILD (7 [25.9%] vs. 25 [52.1%], *p* < 0.05) and a smoking history of 30 pack-years or more (9 [33.3%] vs. 30 [62.5%], *p* < 0.05) (Table 3).

**Table 3:** Characteristics of patients with pneumothorax who received non-surgical treatment as a secondary treatment Abbreviations: COPD, chronic obstructive pulmonary disease; ILD, interstitial lung disease.

	Resolution (Group C) n = 27	No resolution (Group D) n = 48	Test	Statistic	p-value
Age, median (range), years	73 (57–83)	72 (67–82)	Wilcoxon rank-sum	W = 605.5	0.643
Males, n (%)	20 (74.1)	41 (85.4)	Fisher’s exact	—	0.237
>30 pack years, n (%)	9 (33.3)	30 (62.5)	Fisher’s exact	—	0.018
Serum albumin, median (range), mg/dL	4.1 (3.65–4.4)	3.9 (3.5–4.3)	Wilcoxon rank-sum	W = 748.5	0.269
Comorbidity, n (%)					
COPD	10 (37)	22 (45.8)	Fisher’s exact	—	0.479
ILD	7 (25.9)	25 (52.1)	Fisher’s exact	—	0.032
History of pneumothorax, n (%)	11 (40.7)	22 (45.8)	Fisher’s exact	—	0.809

Multivariable analysis showed significant differences in ILD history and smoking history of 30 pack-years or more (Table 4).

**Table 4:** Multivariable logistic regression analysis of factors associated with resolution in patients undergoing non-surgical secondary treatment Abbreviations: CI, confidence interval; ILD, interstitial lung disease; OR, odds ratio.

	OR	95% CI	p-value
Age	1.010	0.970–1.040	0.734
Males	0.722	0.181–2.880	0.645
>30 pack years	3.410	1.100–10.60	0.033
ILD	3.550	1.150–10.90	0.028

**Figure 2:**
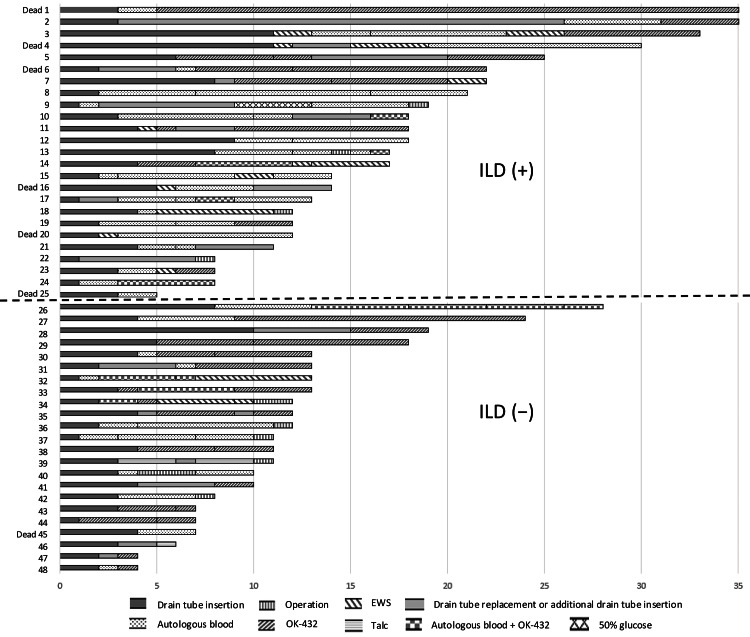
The distribution of treatments and the number of hospital days for the 48 patients with pneumothorax in Group D Abbreviations: EWS, endobronchial Watanabe spigot; ILD, interstitial lung disease.

Additional treatment at each stage was considered if the air leak persisted for over 48 hours after the previous treatment, although timing was largely determined by the treating physician. The distribution of treatments and the length of hospital stay for the 48 patients in Group D are shown in Figure 2.

**Figure 3:**
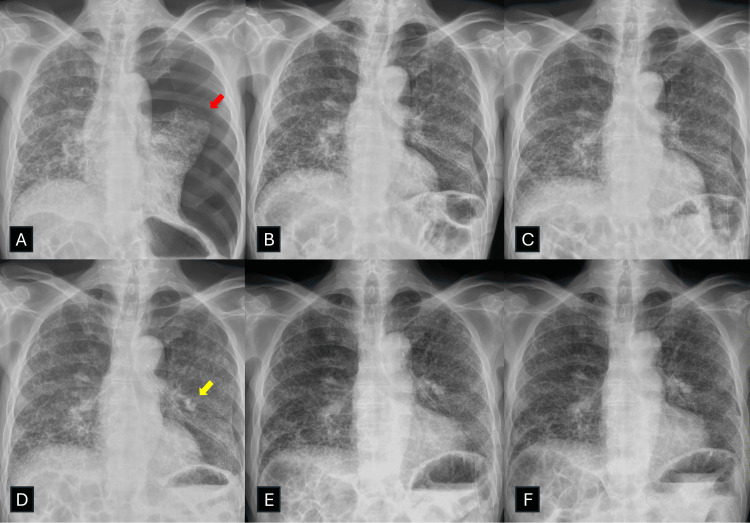
A representative case of protracted ILD-associated pneumothorax in a poor surgical candidate (A) A patient with severe ILD developed a large left-sided pneumothorax (red arrow). (B) After chest tube insertion, the lung partially re-expanded, but a persistent air leak remained. (C) Two sessions of autologous blood-patch pleurodesis failed to control the leak. (D) Placement of an endobronchial Watanabe spigot (EWS) did not result in resolution (yellow arrow). (E) The air leak finally resolved after a third session of autologous blood-patch pleurodesis. (F) The chest tube was subsequently removed, and no recurrent pneumothorax was observed within 24 hours. Abbreviations: ILD, interstitial lung disease; EWS, endobronchial Watanabe spigot.

As a qualitative complement to Figure 2, we present a representative ILD case illustrating a typical non-surgical course in an inoperable patient (Figure 3).

Among the 75 patients with pneumothorax who received non-surgical treatment as a secondary intervention, the resolution rate per treatment was 10/54 (18.5%) for the autologous blood patch and 36/53 (67.9%) for OK-432 (Table 5).

**Table 5:** Resolution rate per treatment in 75 patients with pneumothorax who received non-surgical treatment as a secondary treatment Abbreviation: EWS, endobronchial Watanabe spigot

	Number of resolutions/total number of treatments (%)
Pleurodesis with autologous blood	10/54 (18.5)
Pleurodesis with OK-432	36/53 (67.9)
Pleurodesis with autologous blood + OK-432	5/14 (35.7)
Pleurodesis with talc	1/4 (25.0)
Pleurodesis with 50% glucose	0/1 (0.00)
Surgical treatment	8/10 (80.0)
EWS	3/16 (18.8)
Additional drain tube insertion	2/10 (20.0)
Drain tube replacement	4/14 (28.6)

Among the 48 patients with pneumothorax who did not achieve resolution after the initial secondary non-surgical treatment, the per-procedure resolution rates were 5/49 (10.2%) for autologous blood patches and 19/36 (52.8%) for OK-432 (Table 6).

**Table 6:** Per-procedure resolution rates in 48 patients with pneumothorax that did not resolve after non-surgical secondary treatment Abbreviation: EWS, endobronchial Watanabe spigot

	Number of resolutions/total number of treatments (%)
Pleurodesis with autologous blood	5/49 (10.2)
Pleurodesis with OK-432	19/36 (52.8)
Pleurodesis with autologous blood + OK-432	4/13 (30.8)
Pleurodesis with talc	1/4 (25.0)
Pleurodesis with 50% glucose	0/1 (0.00)
Surgical treatment	8/10 (80.0)
EWS	3/16 (18.8)
Additional drain tube insertion	0/8 (0.00)
Drain tube replacement	2/12 (16.7)

## Discussion

In this study, ILD was a common factor among patients who did not achieve resolution with chest tube drainage alone or by subsequent single non-operative treatments. Few studies have examined the factors influencing the clinical course of pneumothorax at each stage of treatment. Therefore, we examined the risk factors for treatment failure both during primary treatment with chest tube drainage and during secondary non-surgical treatment in patients ineligible for surgery. The present study revealed that patients with a history of pneumothorax or ILD were less likely to achieve resolution with chest tube drainage alone (Group A vs. B) [1], possibly because once a pneumothorax occurs, the ruptured area becomes friable, making the alveolopleural fistula difficult to seal, and because of ILD fibrosis of the lung, which hinders lung expansion [8]. Multivariable analysis also suggested that younger patients were less likely to achieve resolution with chest tube drainage alone. However, this finding may be attributed to prophylactic surgical referral in many of these cases, where the chest tube was retained, and the patients were referred for surgery.

Patients with pneumothorax that does not resolve with chest tube drainage alone are often considered for surgery, as it is the definitive treatment [1,9,10]. However, surgical invasiveness is more likely to exacerbate conditions such as ILD and COPD, making it difficult for patients to undergo surgery [11-14]. Therefore, non-surgical treatments are considered as subsequent treatments. In this study (Group C vs. D), patients with a history of heavy smoking or ILD were less likely to achieve resolution with a single non-surgical medical intervention. This may be explained by smoking-related impairment of cells involved in lung repair and fibrotic changes in ILD that hinder lung expansion [14,15].

In this study, we measured the success rate of non-surgical treatment. At our institution, autologous blood is generally preferred owing to its lower risk of side effects and reduced likelihood of exacerbating ILD compared with OK-432 [16]. The resolution rate per autologous blood patch was 10.2-18.5%, markedly lower than the 78% reported previously [17]. The discrepancy may be explained by the smaller number of patients with ILD in previous studies than in our study. We defined resolution as the absence of recurrence for 24 hours after chest tube removal; however, because previous studies did not clearly define their criteria for resolution, treatment success in those reports may have been determined by the disappearance of the air leak alone. Given the minimal side effects described above, the use of autologous blood adhesion should be actively considered in patients such as those in our study. In contrast, the resolution rate with OK-432 was 52.8-67.9%, which is close to the 63% reported previously [16]. In addition, talc use was low in this study because it was not approved for the treatment of pneumothorax in Japan until recently and because talc is generally not used in patients with an active air leak; it is typically instilled after the leak has resolved to prevent recurrence.

This study had some limitations. First, because this study was not limited to ILD, we did not consider the use of corticosteroids, a history of acute ILD exacerbation, or ILD subtype. Further research investigating these factors is needed. Second, this study lacked external validity because it was a single-center retrospective study. Further multicenter prospective studies, informed by the results of the present study, are warranted. Third, results may have differed if a continuous variable was used; however, a threshold of 30 pack-years or more was used for heavy smoking history, according to the Lung Cancer Screening Guideline 2022 of the Japanese Lung Cancer Society. Fourth, imaging findings were not considered; therefore, the results may have differed depending on the degree of emphysema and fibrosis. Fifth, the doses of drugs and autologous blood used in pleurodesis were not standardized, although in principle, 50-100 mL of autologous blood and OK-432 were used. Sixth, this study did not consider the extent of pneumothorax or patients’ performance status.

## Conclusions

The study findings indicate that patients with a history of pneumothorax or ILD were less likely to achieve resolution with chest tube drainage alone, whereas those with a history of heavy smoking or ILD were more likely to require additional medical treatment. These easily obtainable clinical characteristics can support early risk stratification at the time of admission and help clinicians anticipate a prolonged air leak or the need for additional interventions in patients who are not candidates for surgery. Integrating these predictors into standardized care pathways may optimize consultation timing, monitoring intensity, and patient counseling.
